# The efficacy and prophylactic characteristics of omega-3 fatty acids in experimental gingivitis in rats

**Published:** 2014-02

**Authors:** Nasrin Araghizadeh, Mojgan Paknejad, Mojgan Alaeddini, Bagher Minaii, Mohammad Abdollahi, Reza Khorasanie

**Affiliations:** 1 Dental Research Center, Tehran University of Medical Sciences, Tehran, Iran; 2 Department of Periodontology, Tehran University of Medical Sciences, Tehran, Iran; 3 Dental Research Center, Tehran University of Medical Sciences, Tehran, Iran; 4 Department of Histology, Faculty of Medicine, Tehran University of Medical Sciences, Tehran, Iran; 5 Faculty of Pharmacy, and Pharmaceutical Sciences Research Center, Tehran University of Medical Sciences, Tehran, Iran; 6 Faculty of Pharmacy, and Pharmaceutical Sciences Research Center, Tehran University of Medical Sciences, Tehran, Iran

**Keywords:** Cytokines, Gingival diseases, Histology, Omega-3, Rat

## Abstract

***Objective(s):*** Gingivitis is an inflammatory disease that affects tooth-supporting tissues and is caused by a microbe-immune response. The aim of this study was to evaluate the effects of omega-3 fatty acids on immune system regulation and the prevention and treatment of gingivitis using an animal model.

***Materials and Methods:*** Gingival inflammation was induced by lipopolysaccharide (LPS) injection. Forty adult male rats were divided into four equal groups: 1. Negative control group (sterile saline was injected into gingival tissue followed by oral gavage with saline); 2. Positive control group (LPS injection was followed by oral gavage with saline); 3. Treatment group (LPS injection was followed by oral gavage with omega-3); 4. Prophylactic group (oral gavage with omega-3 was followed by LPS injection). After 24 days, the rats were sacrificed and histological tissue samples were randomly evaluated for the inflammatory tissue changes. Tumor necrosis factor-α (TNF-α) and interleukin-1β (IL-1β) levels were measured by ELISA.

***Results:*** The levels of IL-1β and TNF-α in the prophylactic group and the level of TNF-α in the treatment group were significantly lower than those in the positive control group (*P*<*0.05*). The severity of inflammation was normal, slight, moderate, and severe in the negative control group, prophylactic group, treatment group, and positive control group, respectively. ANOVA was used for the statistical analyses, with *P*<*0.05* regarded as significant.

***Conclusion:*** Prior consumption of omega-3 fatty acids is effective in reducing inflammation in induced rat gingivitis, resulting in a decreased level of biomarkers and fewer destructive effects.

## Introduction

Gingivitis is a common periodontal disease caused by an interaction between host immune responses and the biofilm of pathogenic microorgan-isms in dental plaque (1). Lipopolysaccharide (LPS), an endotoxin produced by gram-negative bacteria, stimulates the metabolism of arachidonic acid. This in turn activates lipooxygenase and cyclooxygenase inflammatory pathways. LPS can also affect macrop-hages, monocytes, fibroblasts and, as a consequence, leads to the production of pro-inflammatory cyto kines, such as TNF-α and IL-1β (2). These are amongst the most important pro-inflammatory cytokines and play a critical role in the destruction of periodontal tissue, alveolar bone, and eventually tooth loss. Also, IL-1β and TNF-α can induce the well as the destruction of connective tissue (1).

Fatty acids are categorized according to the number of carbon-carbon double bonds in their chain: 1- Saturated fatty acids; 2-Fattyacids with only one unsaturated bond; 3- Polyunsaturated fatty acids (PUFAs) with more than one unsaturated bond. Fish oil is the richest source of omega-3 fatty acids. These polyunsaturated fatty acids mainly comprise eiciosapentaenoic acid (EPA) and docosahexaenoic acid (DHA). The main dietary source of PUFAs is cold water fish, such as salmon and mackerel (3).

Omega-3 fatty acids can regulate inflammatory and immunologic reactions if added to the diet. In addition, it has been reported that fatty acids can influence natural killer cells and increase the CD4/ CD8 ratio (4, 5). Human and animal studies have indicated the positive effects of PUFAs in the treatment of a wide range of inflammatory diseases, including rheumatoid arthritis, cardiovascular disea-ses, diabetes, and auto-immune diseases (6-9). But previous research on periodontal diseases has mostly studied the effect of omega-3 on the inflamm-atory mediators, such as arachidonic acid, leukotr-iene B4, prostaglandins, and their products (10-17). Few studies have been conducted on the effect of omega-3 on IL-1β and TNF-α (18, 19). In addition, the local effects of the use of these fatty acids for the treatment of periodontal diseases in both humans and animals have been evaluated, but the results have been controversial (20, 21). On the other hand, any attempt to suppress cytokines can reduce the destruction of periodontal tissues, and there have not been any studies of cytokines and histology. Therefore, considering the positive effects of omega-3 in regulating the immune system and in supper-ssing inflammatory diseases, the present study sought to evaluate the effect of omega-3 on histology and levels of pro-inflammatory cytokines (IL-1β and TNF-α) in rat gingival inflammation.

## Materials and Methods

***Source of materials***

The LPS used was serotype 055:B5 Sigma (USA) diluted in sterile sodium chloride 0.9% (Iran) to a final concentration of 1 mg/ml. The injection volume of LPS was 10 µl in each site. The rats were anaesthe-tized with 0.3 ml/200 g of the mixture comprising of 0.05 mL xylazine 2% and 0.25 ml ketamine hydro-chloride 10% (Rotexmedica, Germany) (16, 22-23).

All injections were performed using insulin syringe (0.3×13 mm) needles. Immunoassay kits were purchased from BioSource International (USA). Omega-3 manufactured by Arnet Pharmaceutical (USA) was purchased from PouraTeb Pharmaceuti-cals (Iran). Each soft gel capsule of omega-3 contained 120 mg of DHA, and 180 mg of EPA, gelatin, glycerin, metilparaben and alpha-tocopherol per milliliter. At the end of the experiment, all rats were anesthetized by an overdose of chloroform inhalation and sacrificed.

***Experimental animals***

The research protocol was approved by the ethics committee of Tehran University of Medical Sciences. The study was conducted according to the ethical guidelines for the care and usage of animals. Forty adult male Sprague Dawley rats (12 weeks old, Pasteur, 180-260 g) were kept under 22ºC and fed standard chow and water in the animal room. The weight of the rats was measured throughout the study period.

***Experimental procedures***

The rats were randomly divided into 4 groups, each containing 10 animals, as follows: 

1) The negative control group received 10 µl (1 mg/1 ml) saline injections under general anesthesia at 6 sites, including the right, middle, and left interdentally papilla of the upper and lower labial aspect of the incisor teeth on 3 consecutive days; the rats were then gavaged with normal sterile saline (60 mg/kg) for 15 days using small sterile plastic tubes. 

2) The positive control group received 10 µl (1 mg/1ml) LPS injections in each site for 3 days under the same conditions as given above; they were then gavaged with normal sterile saline (60 mg/kg) for 15 days. 

3) The treatment group received 10 µl (1 mg/1ml) LPS injections for 3 days under the same conditions as above, and then were gavaged daily with omega-3 (60 mg/kg) for 15 days. 

4) The prophylactic groups were gavaged daily with omega-3 (60 mg/kg) for 15 days, and then injected with 10 µl (1 mg/1 ml) of LPS at each site for 3 days (16, 18). 

***Histological assay***

After the animals were sacrificed, a mesio-distal cross-section of a tissue sample taken from the interdentally central incisor gingival area was dissected in all of the rats in each group. The isolated tissue was fixed in 3 ml of 10% buffered formalin. After histological processing, samples were embedded in paraffin for hematoxylin-eosin staining and then assayed by light microscopy with an original magnification of ×200. Histological grading of the microscopic sections was based on evaluation of inflammatory tissue changes, such as inflammatory cell infiltration, dilated blood vessels, capillary status, densely bundled collagen fibers and, finally, for erosion, edema, and stratified squamous proliferation in the lamina propria and epithelium. The microscopic scores were graded into four categories of inflammatory tissue changes: normal (-), slight (+), moderate (++) and severe (+++) (24). 

***Cytokine analysis***

Tissue sample sections from the upper and lower jaw of the left and right buccal aspect of the incisor teeth were randomly collected and coded. They were then put into sterile plastic tubes and stored at -70 ºC until the concentrations of TNF-α and IL-1β were obtained by immunoassay. Finally, samples were homogenized (1mL buffer + EDTA, 4000 rpm for 40 min) and the total protein content (pg/mg) was analyzed. Bovine serum albumin was used at different concentrations as the protein standard against which the total protein content of the samples was measured. When the bound enzyme was colored, the intensity of the colored products in the original specimen was read by an ELISA reader at λ = 450 nm. The quantitative concentrations of cytokines were expressed as pg of cytokine per mg protein of tissue. 

**Table 1:** The levels of the inflammatory mediators in different groups

Group	IL-1β (pg/mg)	TNF-α (pg/mg)
Negative	233.29± 28.20	169.59± 23.45 (N=10)
Positive	418.24± 40.52*	409.84±110.02* (N=6)
Therapeutic	416.32±19.08*	231.11±26.01 ** (N=9)
Prophylactic	291.75± 24.36**	181.59±27.93** (N=10)

*Significantly different from negative control group at *P< *0.05

**Significantly different from positive control group at *P< *0.05

***Statistical analysis***

Data are expressed as mean ± standard deviation (SD) to ensure the analysis of variance was adequat-ely distributed and to show significant changes among multiple groups, including the negative control, positive control, treatment, and prophylactic groups. 

In order to evaluate the distribution the analysis of variance the Tukey multiple comparison *post-hoc* test was conducted. In addition, ANOVA was used to compare mean values for IL-1β and TNF-α in the different groups. Finally, *P < *0.05 was considered to be significant.

## Results

Five of the 40 adult male rats were lost to unknown causes, without developing a systemic illness. Macroscopic signs of severe inflammation consisting of redness, swelling and edema occurred in the positive control group. Clinical observation of the rats in the prophylactic group showed mild inflammation.

***Cytokines***

**Figure 1:**
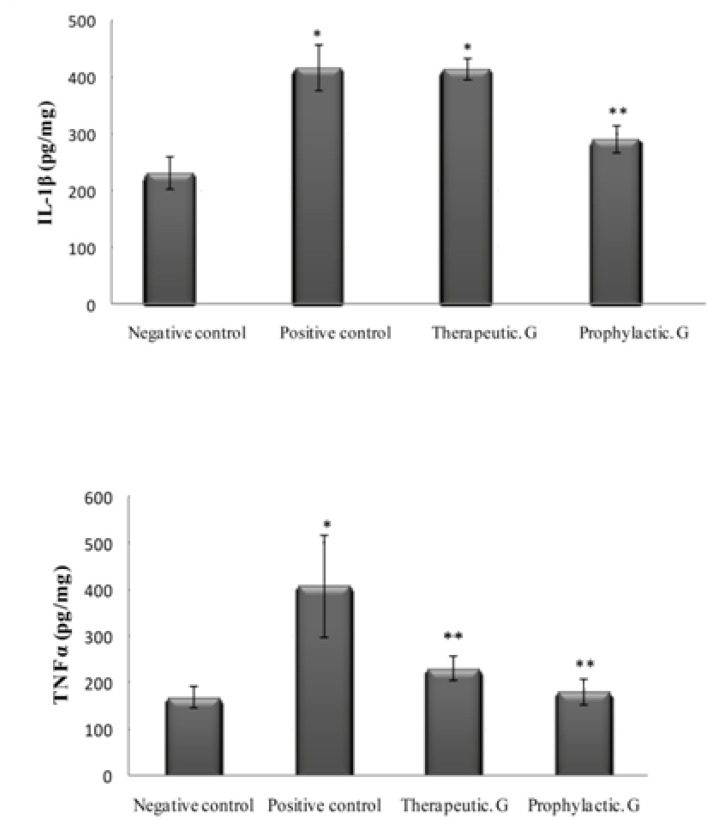
Interleukin-1β (IL-1β) and tumor necrosis factor-α (TNF-α) levels in gingival rat. Values are mean±standard deviation

As shown in Table 1, the levels of IL-1β and TNF-α in the positive control groups were significantly higher than those in the negative control group (*P < *0.05). In addition, the levels of IL-1β and TNF-α in the prophylactic group and the level of TNF-α in the treatment group were significantly lower than those in the positive control group (*P < *0.05), Figure 1. 

**Table 2:** Microscopic scoring of tissue damage

Score	Microscopic criteria
Normal (-)	No changes in the epithelial proliferation, infiltration of inflammatory cells, and dilated blood capillaries.
Mild (+)	Slight inflammatory cells and focally changes in the network vessels, collagen fibers, epithelial prolife-ration, and edema
Moderate (++)	Comparatively frequent inflammatory cells infiltra-tion into epithelium and sub epithelial.
Sever (+++)	Frequent inflammatory cells, proliferation, edema, and erosion in the epithelial and sub epithelium.

***Histology***

The histological assays, as assessed by an expert pathologist, showed that the severity of inflamma-tion was normal, slight, moderate, and severe in the negative control group, prophylactic group, treatme-nt group, and positive control group, respectively, as presented in Table 2. 

**Figure 2:**
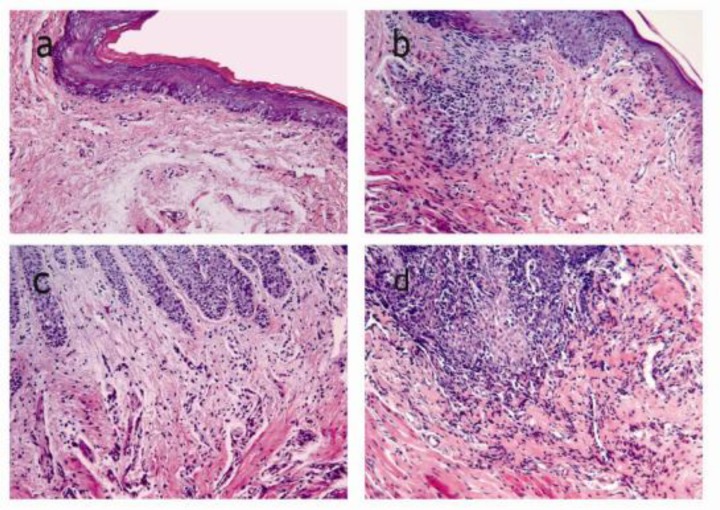
Histological compared to **a:** normal appearance in the negative (Original magnification, ×200).Subepithelial connective tissue including fibroblasts, endothelial cells, gingival epithelial cells and few inflammatory cells **b:** Mild inflammation histological appearance in the prophylactic group.** c:** Moderate inflammation histological appearance in the therapeutic group.** d: **Severe inflammation histological appearance in the positive group

1) Normal scale (-): this view showed a lining of stratified squamous epithelium with a superficial hornified layer. Dense collagen fiber bundles were observed. A few infiltrations of inflamma-tory cells or dilated blood capillaries were also seen.

2) Mild scale (+): this view showed slight inflamm-atory cell infiltration and focal changes in network vessels, collagen bundles, epithelial proliferation and edema.

3) Moderate scale (++): this view showed comparatively frequent inflammatory cell infiltra-tion in epithelial and subepithelial hypervascu-larity, and disruption of collagen fiber bundles. The epithelial proliferation and edema showed comparative multifocality. 

4) Severe scale (+++): this view showed frequent inflammatory cells, hypervascularity and disrup-tion of collagen fiber bundles in the lamina propria. Epithelial proliferation, edema and ero-sion occurred multifocally, as shown in Figure 2.

## Discussion

In the current study, the inflammatory response observed in the periodontal tissues after LPS injection and the development of experimental gingivitis was visible macroscopically after 7 days. All of the animals in the positive control group showed macroscopic signs of inflammation, which was confirmed by histological examination. These findings are consistent with those of the study by Dumitrescu *et al* (22). However, to our knowledge, our study is the first to include histological assay results when reporting the effects of omega-3 supplementation on experimental gingivitis biomarkers (IL-1β, TNF-α).

We found that the levels of IL-1β and TNF-α in the positive control group were higher than those in the negative control group. Similarly, Vardar *et al* reported increases in LTB_4_, PGE_2_, PGF_2α_ and PAF after LPS injection (16, 17). The current study also showed significantly lower levels of IL-1β and TNF-α, in the prophylactic group compared with the positive control group, which may result from reduced arachidonic acid in cell membranes and reduced expression of COX-2 mRNA as a key enzyme of prostaglandin synthesis. Moreover, previous studies have shown decreased production of inflammatory cytokines, such as IL-1, IL-6, and TNF-α due to reduced leukocyte chemotaxis (1, 2, 25).

On the other hand, the present study showed that rats in the treatment and prophylactic groups given omega-3 fatty acids had significantly lower levels of TNF-α than those in groups not given omega-3. TNF-α stimulates endothelial cells, facilit-ating selection and absorption of leukocytes, and finally increases phagocytosis (25). The lower levels of TNF-α may be due to the effect of omega-3 fish oil on TNF-α mediators via decreasing release of macrophages stimulated by bacterial LPS.

Also Kesavalu and colleagues found that rats receiving dietary omega-3 over a period of 22 weeks showed decreased gene expression of TNF-α and IL-1β compared with rats in a control group (19). The findings of Naqvi *et al* are consistent with omega-3 intake having a preventive effect on periodontal disease via an influence on the inflammatory cascade (12).

In the current study, the level of IL-1β in the treatment group was similar to that in the positive control group. Conversely, the results of Vardar *et al* indicated a significant increase in the level of IL-1β in rats that consumed omega-3 fish oil compared with those in the positive control group (18). This discrepancy might be due to the different methods of determining IL-1β in tissue and serum, and differences in omega-3 dosage in the two studies. Therefore, we suggest that future studies should investigate the mechanism of the relationship between IL-1β level and omega-3 fish oil intake.

Another study evaluated the local effects of RvE1 on periodontitis in rabbits and showed that the anti-inflammatory effects of this bioactive product of omega-3 are due to a reduction in the systemic infla-mmatory markers, C-reactive protein and IL-1β (21). 

In the current study the results of histological assays in the prophylactic group indicated slight inflammatory cell infiltration, epithelial proliferation and edema, as seen in the macroscopic view. These histological results suggest that omega-3 may have a stabilizing effect on collagen fiber, and a modulating effect on destruction of gingival connective tissue, by decreasing in levels of IL-1β and TNF-α. IL-1β and TNF-α can induce the production of matrix metalloproteinase (MMPs) as well as the destruction of connective tissue (1). 

Although data from several research publica-tions (10-14, 16-18, 26, 27) suggest that dietary omega-3 improves gingival inflammatory disease in both human and animal studies, data from our study showing a significant decline in levels of IL-1β and TNF-α in the prophylactic group suggest that omega-3 fatty acids may have a potent immune modulator effect and a more preventive than therapeutic effect on the regulation of inflammatory and immunologic reactions.

## Conclusion

Our study supports the notion that prior consumption of omega-3 unsaturated fatty acids, which decreases the level of pro-inflammatory cytokines, is effective in the treatment of experime-ntal gingivitis and decreases the destructive effects of gingivitis. The preventive effects of omega-3 fatty acids seem to result particularly from a reduction in levels of TNF-α. Together with plaque control, diet-ary supplements may be another important factor modulating host response.
